# In Vivo Follicular and Uterine Arterial Indices as an Indicator of Successful Hormonal Stimulation for Inactive Ovaries in Repeat-Breeder Crossbred Dairy Cows Using a Short-Term Progesterone-Based Programme

**DOI:** 10.3390/ani12030292

**Published:** 2022-01-25

**Authors:** Punnawut Yama, Chayanon Yadmak, Molarat Sangkate, Jakree Jitjumnong, Warittha U-krit, Nalinthip Promsao, Napatsorn Montha, Paiwan Sudwan, Raktham Mektrirat, Julakorn Panatuk, Wilasinee Inyawilert, Korawan Sringarm, Chompunut Lumsangkul, Wanaporn Tapingkae, Hien Van Doan, Pin-Chi Tang, Tossapol Moonmanee

**Affiliations:** 1 Department of Animal and Aquatic Sciences, Faculty of Agriculture, Chiang Mai University, Chiang Mai 50200, Thailand; punnawut_y@cmu.ac.th (P.Y.); pondforex39@gmail.com (M.S.); u.warittha@gmail.com (W.U.-k.); paejoojienlt@gmail.com (N.P.); napatsorn1912@gmail.com (N.M.); korawan.s@cmu.ac.th (K.S.); chompunut.lum@cmu.ac.th (C.L.); wanaporn.t@cmu.ac.th (W.T.); hien.d@cmu.ac.th (H.V.D.); 2 Department of Animal Science, National Chung Hsing University, Taichung 40227, Taiwan; j.jakree105@gmail.com (J.J.); pctang@dragon.nchu.edu.tw (P.-C.T.); 3 Lamphun Dairy Cooperative Ltd., Lamphun 51180, Thailand; tamphundairy@gmail.com; 4 Department of Anatomy, Faculty of Medicine, Chiang Mai University, Chiang Mai 50200, Thailand; paiwan.sudwan@cmu.ac.th; 5 Department of Veterinary Biosciences and Public Health, Faculty of Veterinary Medicine, Chiang Mai University, Chiang Mai 50100, Thailand; Raktham.m@cmu.ac.th; 6 Faculty of Animal Science and Technology, Maejo University, Chiang Mai 50290, Thailand; panatuk@gmail.com; 7 Department of Agricultural Science, Faculty of Agriculture, Natural Resources and Environment, Naresuan University, Phitsanulok 65000, Thailand; wilasineei@nu.ac.th; 8 Innovative Agriculture Research Center, Faculty of Agriculture, Chiang Mai University, Chiang Mai 50200, Thailand; 9 The iEGG and Animal Biotechnology Center, National Chung Hsing University, Taichung 40227, Taiwan

**Keywords:** fixed-time artificial insemination, infertile dairy cows, pregnancy rate, preovulatory follicle, ovarian inactivity, ovarian resumption, reproductive failure, vascular index

## Abstract

**Simple Summary:**

Blood supply of female reproductive organs plays an important role in reproductive performance in cattle. Ovarian and uterine arterial indices (vascularised area) from colour Doppler imaging provided important information about ovarian activity, supporting clinical diagnoses and reproductive management decisions in female cattle. However, the information regarding the relationship between reproductive vascular indices and resumption of follicular activity after hormonal stimulation for inactive ovaries in infertile dairy cows is scarce; thus, infertile crossbred dairy cows with inactive ovaries were induced using a 5-day progesterone-based programme. Our results highlighted that repeat-breeder crossbred dairy cows with greater follicular size and follicular and uterine arterial indices underwent a resumption of ovarian activity after hormonal stimulation. Therefore, additional information on follicular and uterine arterial indices that can be helpful in predicting the resumption of ovarian activity after hormonal stimulation in inactive ovary cows can be gained by reproductive vascularisation from colour Doppler ultrasonography.

**Abstract:**

An investigation of vascularity of ovarian and uterine arteries after hormonal treatment for inactive ovaries using the short-term progesterone-based programme had not yet been explored in repeat-breeder crossbred dairy cows. To investigate the in vivo follicular and uterine arterial indices as an indicator of successful hormonal stimulation for inactive ovaries in repeat-breeder crossbred dairy cattle, 59 cows with inactive ovaries were induced with a 5-day progesterone-based protocol. At the completion of hormonal synchronisation, cows were divided into two groups according to the size of the largest follicle (LF) on their ovary: small (≤10.0 mm) and large (>10.0 mm) LFs. Vascularities of LF and uterine artery (UtA) were evaluated using a colour Doppler tool. Cows that presented with large LF had greater follicular and UtA vascular indices (*p* < 0.001) and pregnancy rate (*p* < 0.01) than cows bearing small LF on their ovary. There was a positive correlation (*p* < 0.001) between follicular size and LF and UtA vascular indices. Our findings highlighted that in vivo LF and UtA vascular indices at the completion of hormonal stimulation might be a promising indicator for predicting success in ovarian response to hormonal stimulation for inactive ovaries of infertile crossbred dairy cows.

## 1. Introduction

In dairy production, poor reproductive capacity of heifers and cows leads to economic losses due to deceased production and additional cost on management [1,2]. Repeat breeding is an important reproductive problem that leads to infertility in dairy cows [3]. The high incidence of repeat breeders has been found in 42.3% of dairy herds [4]. Low fertility is partly a result of ovarian dysfunction or impaired function in repeat-breeder dairy cows [5]. Due to the importance of low fertility in dairy cows, a hormonal treatment using a short-term 5-day progesterone (P_4_)-prostaglandin F_2α_ (PGF)-gonadotropin-releasing hormone (GnRH)-based protocol has been widely used to achieve synchronising ovulation of dominant follicle (DF) before fixed-time artificial insemination (FTAI) in dairy cows [6]. Unfortunately, information regarding hormonal treatment in repeat-breeder dairy cows with inactive ovaries using the short-term 5-day P_4_-PGF-GnRH-based programme is scarce.

Under clinical evaluation of reproductive organs using an invasive method, ovarian dysfunction was related to dystrophy of the vascular system in infertile cows [7]. As a non-invasive method, transrectal colour Doppler ultrasonography is considered to be an effective technique for the quantitative evaluation and clinical application of arterial blood flow not only in beef cows [8], but also in dairy cows [9]. Recently, our research group in the study of beef cows [10] and other research teams in the study of mares [11,12] and in dairy heifers [13] demonstrated that the reproductive vascular index, as indicated by the coloured area (pixels and %) of reproductive organ sections from the Doppler sonogram images, is able to indicate the blood supply of the female reproductive system. However, the investigation of vascularity of ovarian and uterine arteries after the hormonal treatment for inactive ovaries using the short-term P_4_-based programme has not yet been investigated in repeat-breeder crossbred dairy cows. To the best of the researchers’ knowledge, a greater knowledge of uterine and ovarian biologies is essential to increase the applied reproduction in female ruminant animals [8,14].

Taken together, these observations led us to hypothesise that the vascular index (coloured area) of the ovary and uterine artery (UtA) would be able to apply as an indicator of successful hormonal induction for ovarian dysfunction in infertile dairy cows. The main objective of the present research was to investigate the in vivo follicular and UtA vascular indices as an indicator of successful hormonal synchronisation for inactive ovaries in repeat-breeder crossbred dairy cows using the short-term (5 days) P_4_-PGF-GnRH-based programme.

## 2. Materials and Methods

### 2.1. Ethics, Animals, Management, and Determination of Ovarian Inactivity

All procedures were approved by the Animal Care and Use Committee of Maejo University (MACUC035A/2564). Mutiparous crossbred Holstein-Friesian (75.0–87.5%) dairy cows from the dairy farms of the Lamphun Dairy Cooperative Limited, Thailand were used in the present study. At the beginning of the study, selected dairy cows were recorded for their lactation number (mean ± standard deviation (SD); 2.7 ± 1.09), body condition score (BCS; 3.1 ± 0.55), insemination number (4.6 ± 1.50), days open (263.4 ± 14.91 days), and mean of daily milk production (13.5 ± 1.01 kg/day). Crossbred dairy cows were reared indoors in a free-stall barn and were milked twice a day. They were fed ad libitum with chopped maize silage and fresh Napier grass (*Pennisetum purpureum*) and supplemented with a commercial concentrate. Clean drinking water and mineralised salt bricks were provided ad libitum.

Before the start of this experiment, all cows were confirmed to have no clinically evident condition of dystocia, retained placenta, metritis or mastitis. A reproductive management procedure that used for inseminating cows before classified as repeated-breeders was insemination of cows based on a visual detected oestrus. Repeat breeder dairy cows that had been defined as a failure to conceive from three or more regularly spaced inseminations in the absence of detectable clinical abnormalities [15] were selected. At the beginning of the study, ovarian ultrasound scans (a 7.5 MHz linear-array ultrasonic probe; HS-1600V, Honda Electronics, Japan) were performed by a single operator to evaluate the structures of the follicles and corpus luteum (CL). A total of 60 selected repeat-breeder dairy cows were classified as having inactive ovaries, indicating ovarian inactivity when a follicular structure less than 5 mm [16] was detected in two consecutive examinations, in the disappearance of a CL or cyst on their ovaries, and no oestrous signs during the 7-day period between the examinations [17].

### 2.2. Hormonal Synchronisation of Repeat-Breeder Dairy Cows with Ovarian Inactivity Using the Short-Term (5 Days) P_4_-PGF-GnRH-Based Programme and FTAI

The short-term 5-day P_4_-PGF-GnRH-based programme of the present study was adapted from a previous study [18]. At the beginning of the hormonal protocol for ovulation synchronisation (day 0), a total of 60 selected repeat-breeder dairy cows with ovarian inactivity were implanted with an exogenous P_4_-releasing device with 1.38 g of P_4_ (controlled internal drug release (CIDR), Eazi-Breed; Zoetis Inc., Mahuhu Crescent, Auckland, New Zealand), concurrent with an injection of P_4_ (50 mg; Steraloids, Inc., Newport, RI, USA), and CIDR inserts were removed from the vagina on day 5. On day 5, all dairy cows received a 250 µg dose (cloprostenol) of PGF (Estrumate, MSD Animal Health, Upper Hutt, Wellington, New Zealand). On day 8, a 10 µg dose (buserelin) of GnRH (Receptal, MSD Animal Health, Upper Hutt, Wellington, New Zealand) was administered concurrently with the FTAI in all dairy cows. The FTAI was performed on day 8 using a single dose of frozen-thawed semen. The completion of hormonal synchronisation period was designated as day 8.

### 2.3. Determination of Resumption of Ovarian Activity in Repeat-Breeder Dairy Cows (with Inactive Ovaries) after Hormonal Synchronisation and Animal Group

On the day of the FTAI (day 8), uterine arterial and ovarian ultrasound scans were performed by a single operator to evaluate the structures of the main arterial vessels supporting the uterus (>5.0 mm in diameter) and the ovarian largest follicle (LF). The relative position and dimensions of the ovarian LF and UtA were sketched on ovarian and UtA charts. Based on the healthy status, a cow was excluded from further experiment if lameness could be identified during the hormonal synchronising period. Thus, repeat-breeder dairy cows with ovarian inactivity (*n* = 59) were divided into two groups according to the LF size on their ovaries [19]: small LF (≤10.0 mm in diameter; *n* = 21) and large LF (>10.0 mm in diameter; *n* = 38), as indicated by the successful hormonal treatment for inactive ovaries.

### 2.4. Evaluation of In Vivo Follicular and Uterine Arterial Indices

To visualise the ovarian LF structure and UtA on the day of the FTAI (day 8), transrectal colour Doppler ultrasonography was performed by a single operator using a 7.5 MHz linear-array ultrasonic probe (HS-1600V; Honda Electronics, Toyohashi, Aichi, Japan) for B mode (grey scale) and Doppler functions (colour flow mapping and power Doppler functions). The amount of cross-sectional tissue areas of ovarian LF (LF ≤ 10.0 mm and LF > 10.0 mm) and UtA contralateral and ipsilateral to the LF ovary in which vascularisation was indicated as vascular areas, as well as being quantified from the Doppler colour sonogram images using the procedures described for beef cows [10] and mares [12]. Briefly, Doppler colour images per pixel density were created using Adobe Photoshop CC software (1990–2017, Adobe system). The Magnetic Lasso tool in Adobe Photoshop CC was applied to outline the power and coloured areas of the ovarian LF and UtA and to subsequently count each area (pixels). The LF antrum was also outlined, and the areas were presented based on distribution of pixels. On the basis of these measurements, thus, ovarian LF vascular indices were expressed as the LF coloured area in pixels (area of the LF colour power Doppler) and the LF coloured area in percentage (area of the LF colour power Doppler [pixels]/LF area without antrum [pixels]). The UtA vascular index was expressed as the UtA coloured area in pixels (area of the UtA colour power Doppler).

### 2.5. Pregnancy Diagnosis

All dairy cows were diagnosed as pregnant using transrectal ultrasonography on day 30 post-FTAI. Uterine ultrasound scans were performed by one examiner to determine the structures of the uterine horns. Dairy cows were defined as pregnant when the embryonic vesicle could be found in the uterine horn.

### 2.6. Statistical Analysis

The animal groups (LF ≤ 10.0 mm and LF > 10.0 mm) were included in the statistical model, as class variables and lactation number, insemination number, and BCS were also included in the statistical model as covariates. Student’s *t* test was used to compare the means of LF and UtA diameters, LF and UtA areas in pixels, and LF area in percentage between dairy cows with LF ≤ 10.0 mm and with LF > 10.0 mm. Data of LF and UtA diameters, LF and UtA areas in pixels, and LF area in percentage are expressed as the mean ± standard error of the mean (SEM). Chi-square analysis was used to compare the percentages of the pregnancy rate (PR) between dairy cows with LF ≤ 10.0 mm and with LF > 10.0 mm. Linear regression was applied to evaluate the linear relationship between ovarian LF diameter and reproductive vascular indices (LF and UtA indices). Linear correlations between ovarian LF diameter and reproductive vascular indices were considered as perfect, very strong, strong, moderate, low, and negligible correlations when the correlation coefficient (r) was 1.00, >0.90, 0.70–0.90, 0.50–0.70, 0.30–0.50, and 0.00–0.30, respectively [20]. A probability of *p* value ≤ 0.05 indicated a significant difference.

## 3. Results

### 3.1. Ovarian LF and UtA

**Figure 1:**
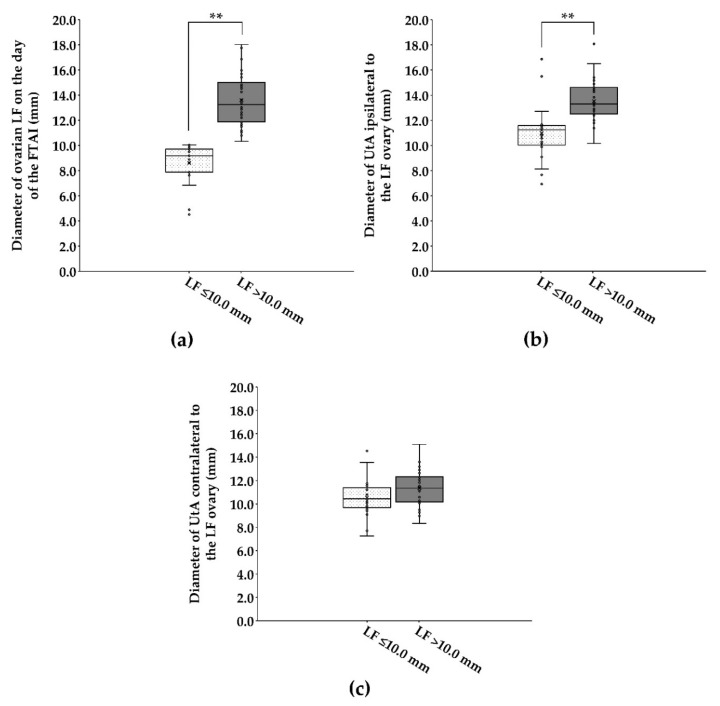
Box plot showing the distribution and the variability of diameters of ovarian LF (**a**) and UtA ipsilateral (**b**) and contralateral (**c**) to the LF ovary on the day of the FTAI in repeat-breeder crossbred dairy cows (with ovarian inactivity) after receiving the short-term P_4_-based programme (*n* = 59). Cows were evaluated and classified according to the LF diameter on the day of the FTAI. LF, largest follicle; FTAI, fixed-time artificial insemination; UtA, uterine artery. ** *p* < 0.01.

The average LF size was greater in cows with LF > 10.0 mm in diameter than in cows with ≤10.0 mm in diameter on their ovary (13.6 ± 0.32 mm vs. 8.6 ± 0.34 mm; Figure 1a). On the day of the FTAI, cows that presented with large LF (>10.0 mm in diameter) had a greater (*p* < 0.001) diameter of UtA ipsilateral to the LF ovary than cows bearing small LF (≤10.0 mm in diameter) on their ovary (13.5 ± 0.25 mm vs. 11.0 ± 0.49 mm; Figure 1b). However, none of diameters of UtA contralateral to the LF ovary differed (*p* > 0.05) between cows bearing small and large LFs on their ovary (10.5 ± 0.36 mm vs. 11.3 ± 0.24 mm; Figure 1c).

### 3.2. In Vivo Follicular and Uterine Arterial Indices

On the day of the FTAI, cows that presented with large LF (>10.0 mm in diameter) had a greater (*p* < 0.001) coloured area of the LF in pixels and in percentage than cows bearing small LF (≤10.0 mm in diameter) on their ovary (6459.7 ± 921.96 pixels vs. 1000.9 ± 136.29 pixels and 6.3 ± 0.33% vs. 2.1 ± 0.18%, respectively). Moreover, cows that presented with large LF (>10.0 mm in diameter) had a greater (*p* < 0.001) coloured area in pixels of UtA ipsilateral (206,388.3 ± 8923.79 pixels vs. 110,608.7 ± 7261.98 pixels) and contralateral to the LF ovary (143,123.7 ± 6721.79 pixels vs. 97,205.5 ± 7999.78 pixels) than cows bearing small LF (≤10.0 mm in diameter) on their ovary.

### 3.3. Association between Ovarian LF Diameter and Reproductive Vascular Indices

**Figure 2:**
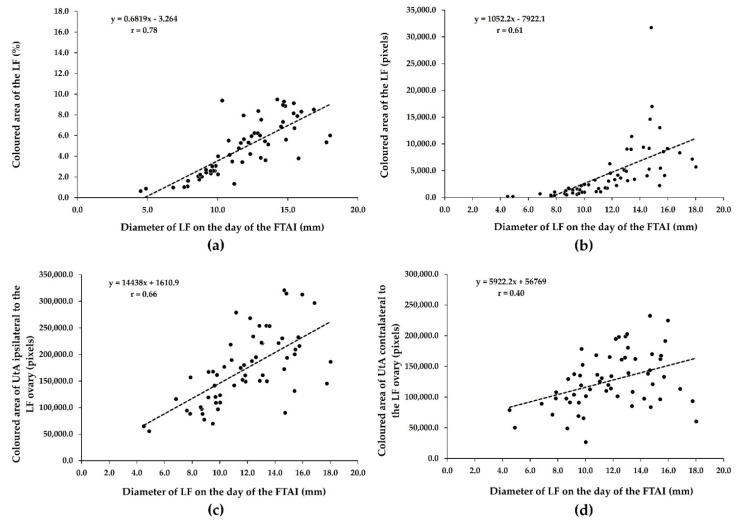
Relationship between ovarian LF diameter and LF (**a**,**b**) and UtA vascular indices (**c**,**d**) on the day of the FTAI in repeat-breeder crossbred dairy cows (with ovarian inactivity) after receiving the short-term P_4_-based programme (*n* = 59). LF, largest follicle; FTAI, fixed-time artificial insemination; UtA, uterine artery.

There was a substantially positive relationship (r = 0.78, *p* = 0.0001) between the diameter of the LF and the coloured area in percentage of the LF on the day of the FTAI (Figure 2a). There was also a moderately positive association between the diameter of the LF (mm) and coloured area in pixels of the LF (r = 0.61, *p* = 0.0001; Figure 2b) and between the coloured area in pixels of UtA ipsilateral to the LF ovary (r = 0.66, *p* = 0.0001; Figure 2c). The diameter of the LF was positively correlated with the coloured area in pixels of UtA contralateral to the LF ovary (minimal correlation; r = 0.40, *p* = 0.001; Figure 2d).

### 3.4. Pregnancy Rate (PR)

**Figure 3:**
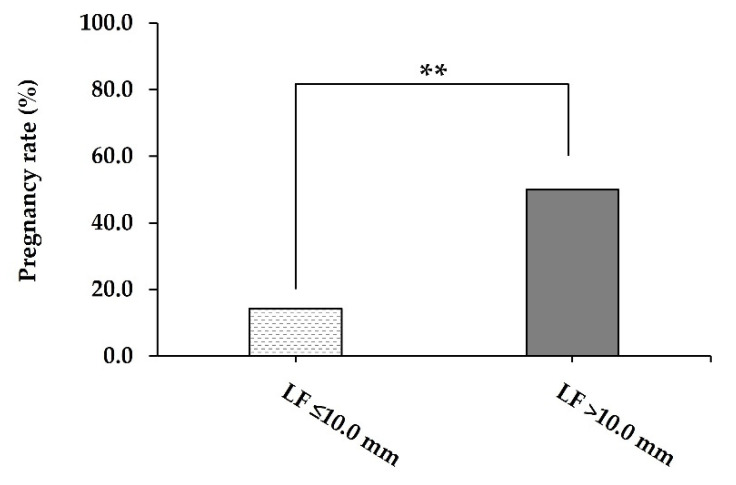
The pregnancy rate (%) on 30 days post-FTAI of repeat-breeder crossbred dairy cows (with ovarian inactivity) after receiving the short-term P_4_-based programme (*n* = 59). Cows were evaluated and classified according to the LF diameter on the day of the FTAI. LF, largest follicle; FTAI, fixed-time artificial insemination. ** *p* < 0.01.

Cows with ovarian inactivity that presented with a large LF (>10.0 mm in diameter) had greater (*p* = 0.007) PR than cows bearing a small LF (≤10.0 mm in diameter) after the end of synchronisation treatment (50.0% vs. 14.3%; Figure 3).

## 4. Discussion

In the present study, repeat-breeder crossbred dairy cows with ovarian inactivity that failed to emerge from the large LF (>10.0 mm in diameter) at the completion of the synchronised ovulation were observed to have lower LF and UtA vascular indices (vascularised area in pixels and %) than those of infertile cows emerging from the large LF on their ovary. To the best of the researchers’ knowledge, this is the first investigation to provide data on in vivo LF and UtA vascular indices as an indicator of successful hormonal synchronisation for inactive ovaries in repeat-breeder crossbred dairy cows using a 5-day P_4_-based protocol. Additional information on follicular and uterine arterial indices that can be helpful in predicting resumption of ovarian activity after hormonal stimulation in inactive ovary cows can be gained by reproductive vascularisation from colour Doppler ultrasonography. The results of the present study support the hypothesis that the vascular index (coloured area) of ovary and UtA can be applied as an indicator of successful hormonal induction for ovarian dysfunction in repeat-breeder dairy cows. Although the information regarding the relationship between ovarian and uterine arterial indices and the resumption of follicular activity after hormonal stimulation in infertile dairy cows is scarce, the blood flow to the preovulatory follicle (POF) is markedly reduced in heat-stressed beef cows (vascularised area in pixels and %) with a synchronised ovulatory cycle [10] and heat-stressed dairy cows (Doppler index) with a spontaneous oestrous cycle [21]. Similar to normally cyclic dairy cattle [22], the vascularity (vascularised area in pixels) of POF and CL is a valuable predictor of pregnancy outcomes in fertile dairy cows. For these female dairy cattle, cows with a high POF vascular index underwent a normal pregnancy [22]. Investigations in normal beef heifers and cows receiving the 7-day Co-Synch + CIDR protocol [23] have observed that blood flow (vascularised area in pixels) of large POF (14.1–17.5 mm in diameter) tended to be greater than small POF (10.8–12.8 mm in diameter). Moreover, the significant increase in vascularity (vascularised area in percent) in POF (>11.5 mm in diameter) suggested that systemic hormonal stimulation may locally induce the ovulatory process [24]. In the cyclic buffalo model, a previous study applied the total blood supply (vascularised area in pixels) of ovarian follicles for predicting the effective hormonal synchronisation that the total blood flow to the DF was greater in females receiving the CIDR-PGF-based protocol than buffaloes receiving the Ovsynch-CIDR-based protocol at oestrous onset [25]. Thus, this strongly implies that ovarian and UtA vascular indices may provide an important indicator to evaluate normal, impaired, and synchronised ovaries in large female ruminants.

At the completion of a hormonal synchronisation period in this study, in vivo LF and UtA vascular indices (vascularised area in pixels and %) were greater in inactive ovary cows with large LF after hormonal stimulation than in cows without large LF on their ovary. The purpose of this study was to gain a better understanding of the resumption of ovarian activity after treatment with hormonal protocol in inactive ovary cows in which the returned growth of ovarian follicles and subsequently POF emergence results not only through hormonal stimulation, but also through a greater blood supply to uterus and follicles. As stated above, in the fertile cow model, Acosta et al. [26] also found that normally cyclic cows had a greater blood flow to POF after hormonal treatment with GnRH. In the present study, administration of the exogenous P_4_ treatment (CIDR) in combination with GnRH and PGF in repeat-breeder dairy cows resulted in a 64.4% (38/59) responsive rate of presented large-sized LF at the completion of the synchronised ovulation. For these responsive cattle, cows had greater follicular and UtA vascular indices on the day of the FTAI. Investigations in beef cows receiving CIDR in combination with GnRH, PGF, and equine chorionic gonadotropin have observed that, during the preovulatory period, POF vascularisation positively correlated with POF growth rates and vasodilator (oestradiol; E2) levels and tended to associate with POF growth rate in ovulating cows [10]. The blood flow of ovarian artery and middle UtA changed after the end of ovarian superstimulation in dairy cows that previously synchronised with CIDR and received follicle stimulating hormone [27]. The significant increase in vascularisations in large LF and UtA ipsilateral and contralateral to the LF ovary, in the present study, suggested that the reproductive blood system may supply enough nutrients, hormones, and growth factors to support follicular growth during hormonal stimulation in inactive ovary cows. The supplies of nutrients, hormones, and growth factors to follicular cells for supporting ovarian activity needs adequate blood flow [28], and maintenance of the follicular vascularisation is important for supporting follicular health [29], maturation, and ovulation [30]. In the present study, inactive ovary cows that returned to ovarian activity as indicated by the emerged large LF and subsequent increase in pregnancy outcome, had greater follicular and UtA vascular indices than cows that failed to emerge large LF after hormonal stimulation. In turn, inadequate ovarian vascularity can act as the trigger that leads to bovine follicular atresia [31]. Although no evaluation of E2 concentrations in inactive ovary cows with large LF after hormonal stimulation was attempted in the present experiment, our results supported the findings of Lopes et al. [32], who reported that follicular size was directly associated with E2 concentrations on the day of artificial insemination (AI) with larger POF related with greater E2 production from POF in dairy cows. A greater growth rate of ovarian follicle increased the E2 production from the granulosa cells of the POF [33]. On a cellular level, ovarian E2 (vasodilator) causes a rapid dilation of blood vessels and subsequently an increase in vascularities of POF [10] and UtA [8,34]. Investigations in dairy cows receiving exogenous E2 have observed that the blood flow to UtA increased in response to E2 treatment [35]. In the present study, the PR was higher in cows bearing a large LF with a greater LF vascularisation than in cows bearing a small LF with a lesser LF on the day of the FTAI. This pattern of results is consistent with the previous study that a greater follicle size on the day of the AI and a high POF blood flow on the day of the AI in dairy heifers that became pregnant compared with non-pregnant heifers [36]. In beef and dairy cattle, the size of the ovarian follicle is not the only indicator that predicts success in ovarian response to hormonal treatment at the end of the synchronisation programme [23,36]. Other parameters such as the correlation between larger POF and high blood flow to POF have confirmed greater rates of establishment of pregnancy [23,36]. Considering the former scenario, POF blood flow on the day of the AI proved to be a better indicator compared to size to evaluate the fate of POF and its effect on conception rate as there was no significant variation in POF diameter among the pregnant, complicated pregnancy, and pregnant dairy cows [22]. This is possibly due to the close relationship between POF diameter and blood flow in which an increase in vascular support to POF is related to greater chances of pregnancy outcome [23,36,37]. Although the ovulation of POF was not evaluated in the present experiment, we speculated, based on previous data, that the ovulating cows had greater blood flow volume compared with the non-ovulating cows [8] and the ovulating POF had the highest percent of the vascularised area in cows [37]. Investigations in dairy cows receiving exogenous PGF or E2 have observed the lower ovulatory synchrony found in the PGF cows compared to the E2 cows [38]. This low response may be explained by the presence of less developed follicles which was indicated by the observed low blood flow of POF [38]. Moreover, under field trial, evaluation of blood flow to reproductive organs using Doppler ultrasonography allows for the determination of highly receptive recipients and could improve pregnancy outcome in embryo transfer programmes [39]. For application of the colour Doppler technology in the field, the relationship between reproductive vascularisation pattern and response to hormonal treatment in the present study might be indicative for practitioners using the colour Doppler tool in the selection of highly responsive cows for insemination after the end of the synchronisation protocol.

Overall, based on our findings, this study directly points to an association between POF growth and reproductive system blood supply regarding an increase in the size of LF in activated ovary cows caused by adequate blood flow to UtA and ovaries for supporting growth of follicular cells after hormonal induction, and subsequently an increase in fertility.

## 5. Conclusions

Follicular and uterine arterial indices from colour Doppler imaging provided important information about the resumption of ovarian activity, supporting clinical diagnoses and reproductive management decisions in infertile dairy cows. Our findings highlighted that a positive correlation between ovarian follicular size and LF and UtA vascular indices (vascularised areas) was observed at the completion of hormonal stimulation in repeat-breeder cows with ovarian inactivity. Repeat-breeder crossbred dairy cows with greater follicular size and reproductive vascular indicators (LF and UtA indices) underwent a resumption of ovarian activity after hormonal stimulation. Therefore, in vivo LF and UtA vascular indices on the day of the FTAI might be a promising indicator for predicting success in ovarian response to hormonal stimulation for inactive ovaries of infertile crossbred dairy cows; however, new and larger studies are necessary.

## Data Availability

The data that support the findings of this study are available from the corresponding authors upon reasonable request.
